# Mapping Knowledge Gaps: A Needs Assessment of Sorghum, Whole Grains Knowledge and Prediabetes Risk

**DOI:** 10.3390/nu18162634

**Published:** 2026-08-12

**Authors:** Andrea Sosa-Holwerda, Surya Raj Niraula, Wilna Oldewage-Theron, Naima Moustaid-Moussa, Leslie Thompson, Oak-Hee Park

**Affiliations:** 1 Department of Nutritional Sciences, Texas Tech University, Lubbock, TX 79409, USA; andrea.sosa@ttu.edu (A.S.-H.); surya.niraula@ttu.edu (S.R.N.); wilna.oldewage@ttu.edu (W.O.-T.); 2 Institute for One Health Innovation and Center of Excellence in Obesity & Cardiometabolic Research, Texas Tech University and Texas Tech University Health Sciences Center, Lubbock, TX 79430, USA; naima.moustaid-moussa@ttu.edu (N.M.-M.); leslie.thompson@ttu.edu (L.T.); 3 Department of Sustainable Food Systems and Development, University of the Free State, Bloemfontein 9301, South Africa; 4 School of Veterinary Medicine, Texas Tech University, Amarillo, TX 79106, USA; 5 Department of Animal and Food Sciences, Texas Tech University, Lubbock, TX 79409, USA

**Keywords:** needs assessment, whole grain consumption, sorghum knowledge, prediabetes risk, adult college students

## Abstract

**Objectives**: The objective of this cross-sectional online survey was to determine university students’ knowledge about whole grain (WG), sorghum, and prediabetes risk. **Methods**: The survey included 45 items to learn about the students’ WG perception, practices, primary nutrition information source, and physical activity level (PAL). Recruitment was carried out using the university’s official email announcement and data was collected through Qualtrics. Two-hundred-and-forty-eight undergraduate and graduate students, aged 18–44 years, completed the questionnaire. **Results**: Sixty-three percent of the students were at risk of prediabetes based on the CDC Prediabetes Risk Test, a recommended screening tool by the National Diabetes Prevention Program. Compared to females, males were at a significantly higher risk of prediabetes and T2DM. Males had a mean ± sd score of 1.70 ± 0.93 versus females (0.64 ± 0.92), (t(246) = −9.07, *p* < 0.001). Participants scored low on overall knowledge (sorghum and WG combined) (4.21 ± 2.04), WG knowledge (2.82 ± 1.03) and sorghum knowledge (3.21 ± 1.23). Despite reporting a high PAL, less than half had a very good health status, with 45.2% of them having overweight or obesity. Social media was the main nutrition information source for 60% of participants. Logistic regression showed that students with obesity had almost four times greater odds compared to those with normal weight (OR = 3.97, 95% CI: 1.66–9.45, *p* = 0.002). **Conclusions**: These results have important public health implications, providing the rationale for our future research on a theory-based nutrition education intervention emphasizing sorghum consumption and metabolic effects on individuals with overweight and obesity, aiming to combat diet-related diseases.

## 1. Introduction

The connection between nutrition knowledge and health literacy is essential for making informed healthy decisions. Concerns have emerged about adults’ health literacy [1]—the capacity to improve or maintain their quality of life by making appropriate decisions regarding disease prevention, health promotion, and health care [2]. The Dietary Guidelines for Americans (DGA, 2020–2025) emphasize incorporating whole grains in the diet [3] for their high dietary fiber and micronutrient contents [4,5], which decrease the risk of developing chronic diseases and risk factors among Americans [3,4].

Despite the strong evidence of health benefits, limited knowledge about whole grains underlines the need for greater awareness to boost its intake [4,6,7], particularly for college students, an essential life stage for forming long-lasting healthy eating habits [6,7]. Compared to all other age groups, college students consume fewer servings of whole grains [8]. Dietary behaviors are further influenced by multiple determinants including nutrition knowledge, food availability, food accessibility, socioeconomic status, and cultural and environmental factors [1,9,10]. Constant exposure to unhealthy foods high in saturated fat, sugar, and sodium results in lower intake of whole grains, fruits and vegetables [1]. For instance, there is scarce knowledge about sorghum, a whole grain with outstanding benefits for human health [11].

Sorghum’s health benefits derive from its high phytochemical content, making it an excellent source of antioxidants [12] and suitable for functional foods [13] which, beyond providing nutrients, contains other health-promoting components [14]. Sorghum’s potential to prevent chronic diseases makes it relevant in the health and nutrition arena, especially in the United States (U.S.) where the prevalence of chronic diseases such as obesity and diabetes is high [15]. Despite its potential, sorghum’s human health benefits remain largely unexplored [13].

Meanwhile, nutrition information is widely disseminated by non-experts through social media, an easy way to access and spread information that is often inaccurate [16,17]. Social media misinformation poses harm to human health, creating barriers to adopting healthy eating habits [18,19]. Moreover, college students struggle to differentiate between reliable and unreliable sources [20].

In 2022, diabetes was the seventh leading cause of death in the U.S. [21] with a rising prevalence among college students [22]. Type 2 diabetes mellitus (T2DM) risk factors may develop given substantial socio-behavioral health changes during college years [23]. Knowledge regarding diseases can motivate people to modify their current lifestyle to lower that risk [22]. Sedentarism, overweight, obesity, and poor eating habits are modifiable risk factors that can delay or prevent T2DM [24].

College students’ unhealthy eating and sedentarism underscore the need to improve knowledge about these risks [22,25]. Thus, our objective was to assess undergraduate and graduate students’ knowledge of (1) whole grains and sorghum, (2) prediabetes risk and its associations with race and weight, and (3) general health, lifestyle behaviors and practices. Additionally, the survey explored students’ perception of and consumption practices of whole grains, and their primary nutrition information source.

## 2. Methods

### 2.1. Study Design

This cross-sectional study was conducted among college students aged 18–44 years at a U.S. southern university. The research was approved by the University’s Institutional Review Board (IRB 2023-749). Informed by the Health Belief Model, the survey instrument was developed based on a literature review on knowledge around sorghum and whole grains, awareness, and prediabetes. To ensure the relevance, flow and clarity of the questions, the survey was reviewed and confirmed by three experts in community nutrition, including a doctoral student. Prior to data collection, the survey instrument was pilot-tested with 5% of the estimated sample size. The final instrument consisted of 45 questions.

### 2.2. Survey Instruments

The survey consisted of five sections collecting information on (1) demographics, including education level, age, gender, and dependents; (2) prediabetes risk, using the validated CDC Prediabetes Risk Test, which included seven questions on diabetes family history, blood pressure, gestational diabetes, and physical activity level (PAL) [26]; participants’ primary nutrition information sources (e.g., health care provider, social media, courses) [27], with multiple selections allowed, (3) whole grains and sorghum, including dietary recommendations (e.g., make half your grains whole grains), and perceptions of their role in disease prevention, with multiple selections allowed based on personal beliefs for reasons to consume whole grains and sorghum; (4) general health-related questions (e.g., health consciousness, health status) [28]; and (5) PAL, using the short-form International Physical Activity Questionnaire (IPAQ) [29]. The questionnaire underwent pilot testing with 17 participants outside the university community. Responses from the pilot test were used to assess the survey’s reliability (Cronbach’s alpha = 0.91). Cronbach alpha did not include the short IPAQ questions or demographic information. It was calculated for knowledge, perception and practice items included in the questionnaire.

### 2.3. Sampling and Recruitment

A minimum sample size of 248 participants was calculated considering a 20% drop-out rate and 80% power. Initially, a total of 413 participants were recruited. Data were collected using an online survey platform (Qualtrics). Potential participants, who were between 18 and 44 years old and enrolled in the university, were contacted through the university’s official email announcement and could complete the survey once. Before completing the survey, participants provided the electronic consent form and those who finished it were entered into a draw for one of five $20.00 Amazon gift cards.

### 2.4. Data Collection and Data Analysis

Data were analyzed using IBM SPSS Statistics 30.0.0 for descriptive and inferential statistics. The results are presented as percentages (categorical variables) and means and standard deviation (SD) (discrete variables). Prediabetes risk score equaled the total points of the 7 questions. For males, 1 point was given for being 40 to 49 years old, having family history of diabetes, high blood pressure, not being physically active and having overweight or obesity. For females, the same criteria were applied, except for the inclusion of an additional question about gestational diabetes diagnosis. A score greater than 0 denotes a risk of having prediabetes and diabetes [26], which was dichotomized as “1” for possible risk and “0” for no risk in the logistic regression analysis. There were no issues of multicollinearity and interaction effects within the explanatory variables included in the final model. The fitness of the model was assessed by the Hosmer and Lemeshow Chi-square test.

An independent *t*-test with a significance level of 0.05 was conducted to compare the prediabetes risk score between females and males and a logistic regression to evaluate the association between prediabetes risk and race, weight status, health consciousness and PAL. Body mass index (BMI) was calculated with self-reported weight and height, where BMI = weight (kg)/[height (m)^2^] [30].

**Figure 1:**
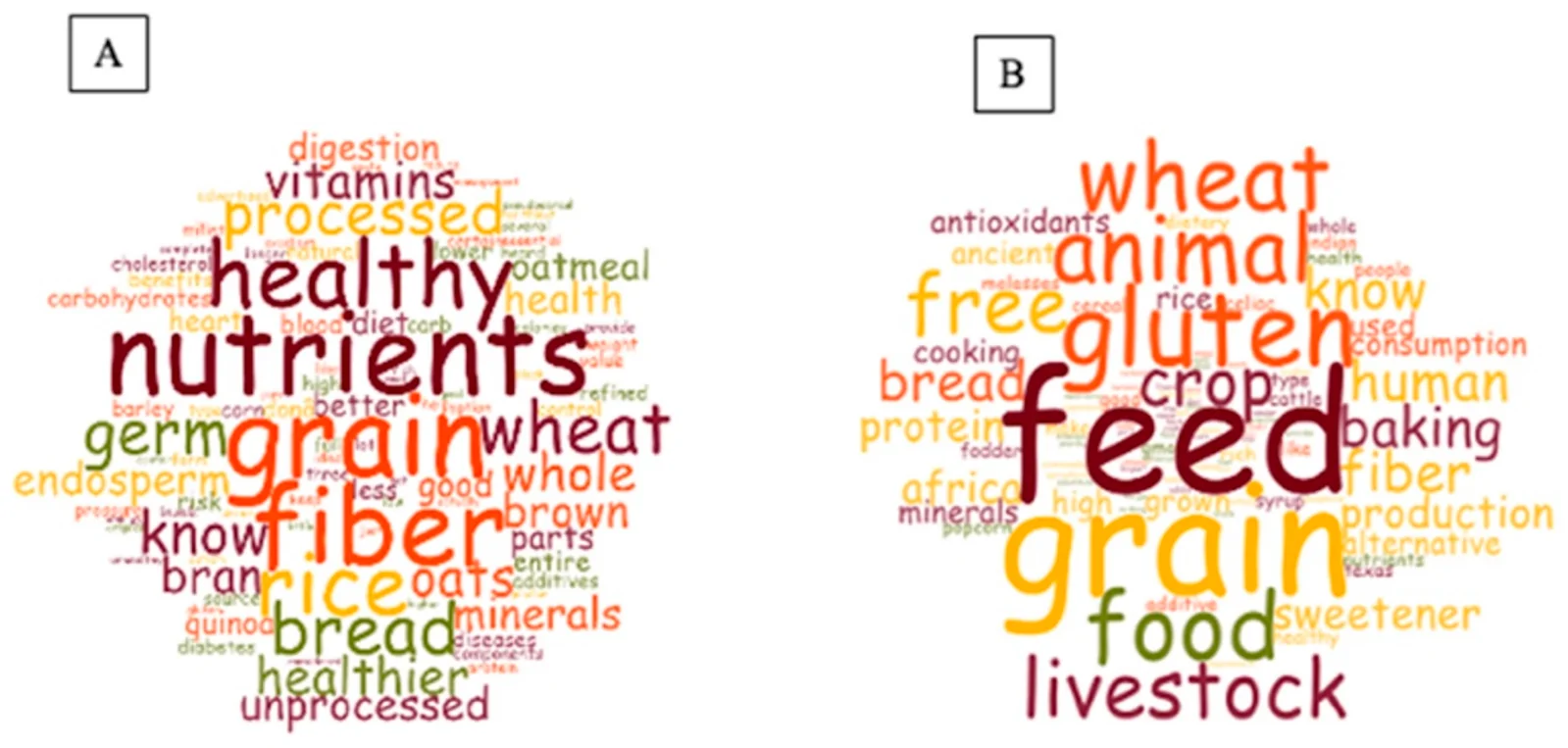
Word clouds for the open-ended questions related to whole grains and sorghum. (**A**) What do you know about whole grains? (**B**) What do you know about sorghum? * 246 participants answered question A and 72 participants answered question B. Figure 1 was created using NVivo 14.0.

Correct answers received 1 point and incorrect or “I don’t know” responses received 0 points. Four questions assessed general whole grain knowledge and three multiple choice questions assessed general sorghum knowledge. One of the sorghum knowledge questions was a multiple-response question (select all that apply) containing three correct options, with each correct option receiving 1 point. Responses were coded as binary (1 = correct or 0 = incorrect or “I don’t know”). Whole grain and sorghum knowledge were scored individually with maximum scores of 4 and 6 respectively. Sorghum knowledge questions were only administered to the 67 participants who indicated that they knew about sorghum; thus, the reported sorghum knowledge score reflects this subgroup. Three questions assessed whole grain perception, and four assessed sorghum perception. The results are presented as percentages reflecting the proportion of participants selecting each response. Furthermore, two questions evaluated sorghum-related practices, also reported as percentages. The participants explained what they knew about whole grains and sorghum in two open-ended questions (Figure 1). The responses were summarized using content analysis following Park et al. (2025) [31] and visualized the most frequently reported words as word clouds using NVivo 14.0. Data from 165 participants were excluded because they had missing responses for more than 10% of the survey items. The final analytic sample included 248 participants.

## 3. Results

Most participants were female (69%), undergraduate (62.1%) and Non-Hispanic White (40.3%), followed by Asian (26.2%), Hispanic (23.8), Black/African (6.9%) and others (2.8%), with a mean age of 25.6 ± 7.40 years (mean ± SD). Sixty-three percent of the participants were identified as being at risk of diabetes exclusively based on the Prediabetes Risk Test provided by the American Diabetes Association (ADA) and the CDC [26], which has been recommended as a screening tool by the National Diabetes Prevention Program to identify those who should undergo further laboratory testing [32]. Males had a statistically significantly higher risk of prediabetes and, consequently, of developing T2DM, with a mean score of 1.70 ± 0.93 compared to females (0.64 ± 0.82 (t(246) = −9.07, *p* < 0.001)) (Table 1).

Of 248 participants, 229 (92.3%) had heard of whole grains, and 203 (81.9%) knew the recommended daily intake. However, more than half of the participants did not know how to identify whole grain foods. The participants perceived that whole grains could prevent heart disease (26.34%), gastrointestinal problems (24.04%), and weight gain (20.37%). Whole grains were viewed as most effective in preventing constipation (22.84%), heart disease (16.16%), diabetes (15.60%), and less effective for weight gain (13.65%), stroke (8.64%), and cancer (5.85%). The main motivations for eating whole grains were to prolong satiety and for losing weight (Table 2).

Participants’ awareness, knowledge, and use of sorghum were limited. Only 27% knew about sorghum. Over 94% were unaware as to whether sorghum was easy to prepare for human consumption, and 54% were unaware of its use in the food industry. Nonetheless, 65.7% perceived sorghum healthy for human consumption (Table 2).

Figure 1 presents word clouds for the two opened-ended questions after content analysis. The more frequently a word appears, the larger it is. The participants associated whole grains with nutrients, fiber, health, rice and bread. In contrast, sorghum was mainly associated with animal feed, livestock, and being gluten-free.

The participants self-reported their physical activity as mostly high (63.7%) and moderate (20.6%), and rated their health status as very good (42.7%) and fair (42.3%). However, 50% were occasionally concerned about healthy diets. Interestingly, 45.2% had overweight or obesity. Although the state-level prevalence has been reported as 34.4%, differences in population characteristics between the adult population and university students should be considered when interpreting these findings [33] (Table 2), consistent with reported obesity demographics [34].

A logistic regression was conducted to assess whether age, race/ethnicity, weight status, health consciousness and physical activity were associated with prediabetes risk based on the CDC Prediabetes Risk Test. The criteria used to select the variables for the logistic regression correspond to the study’s objectives to examine the association by age, race/ethnicity, weight status, health consciousness and physical activity. Moreover, these are considered risk factors of diabetes [35]. The overall model was statistically significant [χ^2^(12) = 59.41, *p* < 0.001]. Students aged 30 years or younger had significantly lower odds of being associated with prediabetes risk based on the CDC Prediabetes Risk Test compared to those aged 30 years or older (B = −1.37, *p* = 0.004, OR = 0.26, 95% CI [0.10, 0.64]). Additionally, students with obesity had significantly increased odds of prediabetes risk based on the CDC Prediabetes Risk Test compared to those with normal weight (B = 1.38, *p* = 0.002, OR = 3.97, 95% CI [1.66, 9.45]), indicating that students with obesity are almost four times more likely to be associated with risk of diabetes compared to students with normal weight (Table 3).

## 4. Discussion

This study’s main findings were that (1) 63% of participants were at risk of prediabetes; (2) the participants had a significant knowledge gap on whole grains and an even greater gap related to sorghum; (3) although over 63% self-reported high PAL, only 42.7% reported very good health status; and (4) social media was the main nutrition information source. Self-reported data was obtained due to the nature of the needs assessment.

### 4.1. Prediabetes Risk

It is documented that 70% of college students gain weight during their freshman year, with a tendency to continue this trajectory through senior year. Poor lifestyle and eating behaviors may increase health problems to those already at risk [36]. This research identified prediabetes risk among college students. Our results are based exclusively on the CDC Prediabetes Risk Test, and no biomarkers were measured as confirmatory lab tests (e.g., HbA1c, fasting glucose, or oral glucose tolerance test). Gamston, C.E. et al. (2020) demonstrated that, compared with the Prediabetes Screening Test, the CDC Prediabetes Risk Test had greater sensitivity and specificity for detecting prediabetes as a screening tool [32]. Male participants were at a significantly higher risk of developing T2DM compared to females. These findings are consistent with sex-related predisposition risk [37]. Confirmed by Logue et al. (2011) [38], T2DM is diagnosed among men across all ages. Another study found that 85.7% of male students were at intermediate risk of T2DM compared to the 44.4% intermediate risk of females [23]. These findings underscore the importance of using screening tools to identify college students who are at risk and suggest that sex-specific differences should also be considered when developing and applying prevention strategies. Our results showed that Hispanic participants had lower odds of being classified as at risk of diabetes compared to their Non-Hispanic White counterparts. This finding differs from the national trends showing a higher prevenance of T2DM among Hispanic populations. However, the sample in this study includes a single university and does not represent the broader U.S. population. Therefore, this finding may reflect a characteristic specific to this university sample. Moreover, we found a 45.2% prevalence of obesity or overweight in our sample, higher than the prevalence of obesity in Auburn University (31%) and the University of Florida (33.2%) [39]. This could be attributed to the institutions’ geographic locations, demographics of the enrolled students (e.g., age, gender, ethnicity), and the variety and availability of the food options offered to students. Research shows that excess body fat can lead to T2DM, with the risk increasing proportionally as BMI increases because obesity is also associated with insulin resistance and inflammation, two other risk factors for T2DM [40,41].

Given the global rise in T2DM and its close association with the increasing prevalence of obesity, there is an urgent need to address this serious public health issue [40,42]. In our study, age and weight were significantly associated with prediabetes risk, based on the CDC Prediabetes Risk Test, highlighting the need for early prevention [43]. Hence, prediabetes risk early in life should be taken seriously, especially when overweight and obesity are concurrent to poor dietary habits, and they are positively correlated [44].

### 4.2. Whole Grain and Sorghum Knowledge

Limited knowledge of whole grains, lack of understanding of their health benefits, and not being able to recognize whole grain foods hinder the motivation to eat them [45]. Furthermore, doubts about the health benefits of carbohydrate-rich foods seem to discourage the overall grain consumption [45]. Our findings showed that most participants had heard of whole grains. However, over half of them scored low on whole grains and sorghum knowledge, suggesting that being familiar with the term “whole grains” does not imply sufficient knowledge and understanding of them [46].

The word clouds showed that while participants associated whole grains with nutrients, fiber and health, they also used terms like “processed” and “unprocessed”, suggesting knowledge gaps about what constitutes a whole grain and its characteristics. Ugunesh et al. (2023) found that over 80% of their participants lacked whole grain knowledge, a major barrier for consumption [47]. In agreement, the participants in our study linked whole grains primarily with rice and bread. Therefore, we can infer that their whole grain consumption is limited to these two products. The participants indicated limited understanding of sorghum’s use as human food and nutritional value, associating it mainly with animal feed, livestock and gluten-free, highlighting the need for nutrition education to promote sorghum consumption and its potential health benefits [31].

Nutrition knowledge facilitates compliance with the 50% whole grain intake recommendation [3,4,6]. Most Americans (98%) and college students do not meet the recommended whole grain intake [8,48], emphasizing the need for nutrition education to improve eating behaviors [4]. However, nutrition knowledge is one of the many factors influencing dietary intake; for example, economic stability, medical conditions, culture, and education are other determinants that impact food choices [49]. As expected, sorghum scored low in knowledge, likely due to its limited role in U.S. diets, low commercialization and scarce consumer exposure [50]. Our results indicated that participants did not know about sorghum applications in the food industry or its suitability for human consumption; this could be ascribed to its main use as biofuel and animal feed in the U.S. [15,31,51,52].

Constant exposure to healthy foods is important, as it can augment the acceptance and intake of new foods, especially when they are not part of the diet [53]. Low sorghum consumption can be attributed to its low exposure. In Asia and Africa, sorghum acceptance and knowledge are greater [54] due to programs aimed at educating consumers, farmers, health personnel, and the food industry about sorghum’s health benefits [54], indicating that effective nutrition education intervention can successfully enhance sorghum consumption and acceptance [4,54,55].

Whole grain consumption is associated with the prevention of chronic diseases and gastrointestinal disorders [56]. Our results revealed an interesting discrepancy between perception and whole grain effectiveness and the motivation for consuming them. The participants acknowledged that whole grain consumption can help to prevent heart disease and T2DM. However, their primary motivation for consumption was satiety and weight loss, not disease prevention. This discrepancy may arise from the association between whole grains’ dietary fiber and weight loss [57,58].

Some of the main barriers to consuming whole grains are price and availability, especially among college students [4,9]. Foster, Beck confirmed that when the prices of whole grain foods and refined grains were the same and both were available at the dining facility, whole grain consumption among college students increased [4], suggesting that affordable pricing and availability play a crucial role in promoting whole grain consumption. Moreover, over 50% of our participants reported being occasionally concerned or not concerned about having a healthy diet.

People often perceive time constraints and prioritize social life and family over healthy eating [59]. Aligned with this, Pelletier and Laska revealed that for adults, lack of time is a common barrier to healthy eating, resulting in poor adherence to dietary recommendations [60]. Therefore, when considering healthy eating, issues such as time, cooking skills and food preferences should be considered [60].

### 4.3. Physical Activity and Health Status

Health issues and metabolic disorders increase when the recommended amount of physical activity is not met. Hence, awareness about unhealthy lifestyles is essential for public health and disease prevention [36]. Most participants self-reported high physical activity; according to the short IPAQ, they either engaged in vigorous physical activity ≥ 3 days reaching ≥ 1500 metabolic equivalents of task (MET) or a combination of walking, moderate and vigorous activity ≥ 3000 MET [61]. Despite the high PAL reported by the participants, almost half of them were classified as having obesity or overweight. Body weight and obesity are influenced by multiple determinants including diet, stress level, sleep habits, genetics, family history, health conditions, medications, and age, which may have contributed to the participants’ weight status regardless of their PAL [62,63]. Moreover, access, income, and environmental factors also contribute to the complexity of obesity [63]. Dietary intake was out of the scope of this study; no association with diet and weight status was determined. Although objective measures such as pedometers and accelerometers generally provide more precise estimates of PAL, the use of validated self-reported tools like the IPAQ can also be used to estimate PAL [64,65]. Less than half of our participants reported having a very good health status. This finding shows that while physical activity is important, it may not be the sole determinant of perceived health status. This finding is supported by Caramenti and Castiglioni, who revealed that participants’ health perception was also associated with mental health, cognitive functioning, medications, and BMI [66].

### 4.4. Nutrition Information Sources

Science-based nutrition information sources are essential for making informed dietary choices and improving health [67]. Our findings illustrated the overwhelming reliance on social media for nutrition information (Table 2). Previous studies have shown that much of this information is provided mainly by non-experts, with just 5% authored by professional nutritionists [68]. Moreover, the literature has demonstrated that exposure to fad diets, unhealthy eating and fake body images on social media are factors that may foster overly restrictive eating behaviors among adults [69]. Although social media can facilitate recipes and nutrition information, Law and Jevons reported that even students in nutrition and dietetics majors indicated that social media affected their eating habits, posing a risk factor for developing eating disorders [70], whereas interventions incorporating nutrition education conducted by experts in the field lead to successful healthy weight loss [71].

### 4.5. Sorghum and Obesity

In this study, a notable percentage of students had overweight and obesity. Students with obesity are almost four times more likely to develop prediabetes compared to those with normal weight, based on the CDC Prediabetes Risk Test, consistent with the stablished role of body weight in metabolic health [72]. Previous studies have shown that the content of phytochemical compounds in sorghum fight against oxidative stress [12,73], thereby lowering lipid accumulation and inflammation markers, both associated with obesity and overweight [74], suggesting the potential role of sorghum in obesity prevention [75].

## 5. Strengths and Limitations

This study’s limitation is its reliance on participants’ self-reported height, weight, physical activity and health status. Self-reported measurements are susceptible to misreporting, potentially affecting the reliability of the prediabetes risk assessment. Although the CDC Prediabetes Risk Test is a validated self-reported screening tool, reporting bias may have influenced the participants’ responses and, consequently, the estimated prediabetes risk and the regression analyses using these variables. In the logistic regression, some categories included relatively few participants, which may have affected the stability of some estimated odds ratios. Additionally, the study’s generalizability to the U.S. population is limited because participants were recruited from a single university. Future studies should incorporate more objective measurements and biochemical markers such as HbA1c and fasting glucose to confirm prediabetes or diabetes status and enhance data accuracy. Due to the nature of the cross-sectional study, causality cannot be inferred. Despite its limitations, this study addresses an important public health issue, by examining prediabetes risk and its modifiable risk factors such as physical inactivity and high BMI among university students. This study also provides relevant information on whole grain and sorghum knowledge in a vulnerable population, helping identify nutrition educational gaps, informing future nutrition interventions for the early detection of diabetes and other chronic diseases, especially in an environment where the availability of nutrient-dense foods like whole grains is limited.

## 6. Conclusions and Implications

This study provides valuable insights into gaps, guiding the direction of future research and intervention strategies. Prediabetes risk was measured exclusively using the CDC Prediabetes Risk Test, which may help to identify individuals at risk of diabetes and its comorbidities, supporting diabetes prevention efforts. Together, the assessment of whole grain and sorghum knowledge, prediabetes risk, and lifestyle behaviors provides a more comprehensive understanding of the target population and identifies areas for prediabetes and diabetes prevention as well as areas for future nutrition education interventions. We identified a significant gap in whole grain knowledge and a greater one on sorghum knowledge, including its use as human food and its health benefits. Social media as a primary nutrition information source amongst the target population underscores the need for expert-led nutrition education to promote informed, science-based decisions and healthier lifestyles. These findings highlight the need for tailored nutrition intervention to improve the overall knowledge about whole grains, specifically sorghum, to assess its potential health effects. This study laid the foundation and provided the rationale for our future research on a theory-based nutrition intervention for sorghum consumption and its metabolic effects in individuals with overweight and obesity, with the ultimate goal of promoting the “food is medicine” concept and reduce diet-related diseases.

## Figures and Tables

**Table 1:** Prediabetes risk scores by sex among college students (*n* = 248).

Gender	Mean (SD)	*t*	*df*	*p* Value
Males	1.70 (0.93)			
Females	0.64 (0.82)			
*t*-test		−9.07	246	<0.001

**Table 2:** Knowledge scores, perception, and motivation for consuming whole grains and sorghum; self-reported PAL, health status, diet concern, and primary source of nutrition among college students (*n* = 248).

Variable		
**Knowledge**	**Mean**	**SD**
Total whole grain knowledge (0–4)	2.82	1.03
Total sorghum knowledge (0–6) ^a^	3.21	1.23
**Whole grain knowledge**	***n***	%
Have you heard of whole grains before?		
No	19	7.7
Yes	229	92.3
A food is whole grain if there’s a logo/picture on the package		
Incorrect	111	44.8
Correct	137	55.2
A food is whole grain if whole grain is mentioned on the package		
Incorrect	117	47.2
Correct	131	52.8
Whole grain intake recommendation		
Incorrect	45	18.1
Correct	203	81.9
**Perception about whole grain ^b^**		
Whole grain perception in disease prevention ^b^		
Heart disease	172	26.34
Gastrointestinal problems	157	24.04
Type 2 diabetes	172	26.34
Weight gain	133	20.37
None of the above	19	2.91
Whole grain perception in effectiveness of disease prevention ^b^		
Disease	Least efficacious (***n***) %	Most efficacious (***n***) %
Stroke	9 (7.50)	31 (8.64)
Diabetes	3 (2.50)	56 (15.60)
Heart disease	1 (0.83)	58 (16.16)
Cancer	30 (25.0)	21 (5.85)
Weight control	11 (9.17)	49 (13.65)
Constipation	4 (3.33)	82 (22.84)
Don’t know	62 (51.67)	62 (17.27)
Motivations for eating whole grain ^b^	***n***	%
Lose weight	134	22.41
Keep full longer	189	31.61
Like the taste	96	16.05
Served at home	79	13.21
Cheap to buy	77	12.88
Other	23	3.85
**Sorghum knowledge ^a^**		
Do you know about sorghum?	***n***	%
No	181	73
Yes	67	27
Sorghum can be consumed by people		
No	8	11.94
Yes	59	88.06
What can sorghum be used for		
Diabetes management		
No	27	40.30
Yes	40	59.7
Feeding babies		
No	61	91.04
Yes	6	8.96
Losing weight		
No	40	59.70
Yes	27	40.30
Sorghum is high in Iron		
No	51	76.12
Yes	16	23.88
Sorghum is high in Calcium		
No	57	85.07
Yes	10	14.93
**Perception about sorghum**		
Sorghum is healthy for human consumption	***n***	%
No	18	7.3
Yes	163	65.7
Don’t know	67	27
Sorghum is animal feed		
No	27	10.89
Yes	65	26.21
Don’t know	156	62.90
Sorghum is used in food industry		
No	6	2.42
Yes	108	43.55
Don’t know	135	54.03
Sorghum is easy to prepare for human consumption		
No	3	0.45
Yes	12	5.2
Don’t know	233	94.35
**Physical Activity Level (PAL) ^c^**	***n***	%
Low	39	15.7
Moderate	51	20.6
High	158	63.7
**Health Status**	***n***	%
Excellent	23	9.3
Very good	106	42.7
Fair	105	42.3
Poor	11	4.4
Don’t know/not sure	3	1.2
**Concerned about a healthy diet**	***n***	%
Not concerned	19	7.7
Occasionally concerned	124	50
Very concerned	105	42.3
**Body Mass Index (BMI)**	***n***	%
Underweight	9	3.6
Healthy weight	127	51.2
Overweight	62	25
Obesity	50	20.2
**Source of nutrition information ^b^**	***n***	%
Social media	169	67.30
Health care provider	133	52.8
Advise from friends	103	40.70
Other	65	23.70
Courses	52	19.60
TV shows	30	11.10

For whole grain knowledge and sorghum knowledge, correct answers received 1 point and incorrect or “I don’t know” responses received 0 points. ^a^ Sorghum knowledge only included respondents who reported knowing about sorghum (n = 67). The effectiveness of whole grains of disease prevention was measured in a scale from 1 to 5, where 1 = least efficacious, 3 = neutral and 5 = most efficacious. ^b^ Multiple options were allowed to be selected. ^c^ Physical Activity Level: Low = Not meeting the criteria of moderate or high PAL; Moderate = high physical activity for 3 or more days and/or walking of at least 30 min/day, or a combination of walking and moderate- or high-intensity activities and reaching at least 600 MET min/week; High = physical activity for at least 3 days and reaching at least 1500 metabolic equivalents of task (MET) or they engaged in a combination of walking, moderate and vigorous activity of minimum 3000 MET.

**Table 3:** Logistic regression predicting odds for diabetes risk association by age, race/ethnicity, weight status, health consciousness, and physical activity (*n* = 248).

Variable	B	*p* Value	OR (95% CI)
Age group (<30 vs. >=30)	−1.37	0.004	0.26 (0.10–0.64)
**Weight status (Ref. normal weight)**		0.017	
Overweight vs. normal weight	0.56	0.139	1.75 (0.84–3.65)
Obesity vs. normal weight	1.38	0.002	3.97 (1.66–9.45)
Underweight vs. normal weight	0.32	0.699	1.37 (0.27–6.90)
**Physical activity (Ref. High PAL)**		0.513	
Low vs. high	0.47	0.324	1.6 (0.63–4.06)
Moderate vs. high	0.36	0.412	1.44 (0.61–3.41)
**Health conscious (Ref. Very health conscious)**		0.378	
Very vs. not at all	−0.96	0.186	0.38 (0.09–1.59)
Reasonably vs. not at all	−0.59	0.363	0.55 (0.15–1.99)
**Race/ethnicity (Ref. Non-Hispanic White)**		0.000	
Asian vs. Non-Hispanic White	1.35	0.001	3.87 (1.69–8.87)
Black vs. Non-Hispanic White	−0.46	0.413	0.63 (0.21–1.89)
Hispanic vs. Non-Hispanic White	−0.75	0.045	0.47 (0.22–0.98)
Other vs. Non-Hispanic White	−0.32	0.756	0.73 (0.01–5.41)
Constant	1.74	0.037	

Significance level at 0.05, −2 Log likelihood = 266.61, Hosmer and Lemeshow Chi-square = 6.09, *p* = 0.637.

## Data Availability

The raw data of this article will be made available by the authors upon publication and request.
